# The effects of factors on the motivations for knowledge sharing in online health communities: A benefit-cost perspective

**DOI:** 10.1371/journal.pone.0286675

**Published:** 2023-06-12

**Authors:** Pei Wu, Runtong Zhang, Jing Luan

**Affiliations:** School of Economics and Management, Beijing Jiaotong University, Beijing, China; SGH Warsaw School of Economics: Szkola Glowna Handlowa w Warszawie, POLAND

## Abstract

Online health communities (OHCs) provide knowledge for users, enabling conversations across a broad range of health topics. The development of OHCs depends on users’ motivations to share health knowledge. Yet little literature has explored how perceived benefits and costs affect users’ motivations for sharing both general and specific knowledge. Based on social exchange theory, we propose a research model that comprises intrinsic benefits (sense of self-worth, satisfaction), extrinsic benefits (social support, reputation, and online attention), cognitive cost, and executional cost to investigate the effects of these factors on users’ motivations for general and specific knowledge sharing. We compare the different effects of these factors on users’ motivations for knowledge sharing. Results demonstrate positive effects of intrinsic and extrinsic benefits on users’ motivations for general and specific knowledge sharing. Differences exist in the negative effects of cognitive and executional costs on users’ motivations for general and specific knowledge sharing. This study contributes to promoting the enrichment of online health knowledge and provides implications for the development of OHCs.

## 1 Introduction

The healthcare industry is one of most significant sectors in daily life. Information and communications technologies are transforming how people learn about health knowledge [1]. With the help of these technologies, sharing others about health knowledge, treatment experiences, and related diseases feelings is now much simpler than ever before [2]. The Pew Research Center reports that 26% of adult internet users have read about health experiences, and 72% of adults have searched online for a variety of health-related issues, with specific diseases and treatments being the most sought-after queries [3]. Online health communities (OHCs) are places where people can share knowledge about many aspects of health. OHCs are designed for healthcare professionals, including physicians, nurses, and other healthcare practitioners, to connect, collaborate, and share knowledge related to their field of expertise. OHCs run by Internet companies and provide a platform for professional networking, continuing education, and knowledge exchange among healthcare professionals and patients. Patients can seek health advice and information from physicians and other healthcare professionals by posting questions and receiving responses. Physicians provide medical assistance by answering questions, providing professional knowledge, and interacting with patients. Additionally, patients can also communicate with other patients in OHCs, sharing treatment experiences, emotional experiences, and lifestyle tips, and providing emotional support. OHCs provide a convenient, real-time, and highly interactive platform that promotes knowledge sharing, mutual assistance, and interaction between patients and healthcare providers [4–6]. Health knowledge sharing is one of the essential elements for the ultimate success of OHCs [7]. Understanding why some users share knowledge while others do not is the challenge for managers of OHCs [8]. Thus, exploring users’ motivations for knowledge sharing is crucial to OHCs development.

Knowledge sharing is an action of communication that takes place voluntarily in a social context [9]. Previous studies have examined what drives knowledge sharing in online communities [10–12]. Users in OHCs are typically urged to actively engage in communication activities. Users are motived to exchange in-depth health knowledge by the intrinsic and extrinsic benefits [13]. Based on social exchange theory, users contribute knowledge in online communities when the perceived benefits of their participation behavior exceed the costs [14]. Executional costs are the time and energy spent by users to share knowledge, whereas cognitive costs are the effort of identifying and screening knowledge in OHCs [15]. Users are hesitant to share knowledge because they are unsure of its applicability to a specific discussion [16]. General and specific knowledge are the two categories of knowledge in OHCs [7, 15]. General knowledge refers to free public health education and professional information, whereas specific knowledge pertains to personal health experiences and treatments. In the context of OHCs, general knowledge comprises the word-of-mouth of physicians, professional effects, and prices. Sharing general knowledge can improve physicians’ performance to help patients with chronic diseases, and reduce health disparities between urban and rural areas [5, 17, 18]. Users’ information on experiences, online consultations, medical interventions, and health restoration related to information. Sharing specific knowledge is especially important for people who suffer from similar conditions [15]. Health professional knowledge is specific in the context of OHCs. It is a research gap that the motivations for sharing both general and specific knowledge in OHCs have not been sufficiently investigated in previous studies.

In OHCs, knowledge offers users with chronic diseases social support [19]. Social support has a positive impact on the motivation to share knowledge [15]. People will gain attention in online communities if others value their knowledge. A significant extrinsic driver of knowledge sharing is online attention [20]. Online attention reflects users’ acquisition of social capital in OHCs [21]. Another dimension of social capital is reputation. Reputation has a positive impact on users’ intentions to share knowledge [13]. In addition, a high sense of self-worth among users makes them more willing to communicating [22]. Users are satisfied with the engagement of sharing knowledge when the anticipated benefits are matched to the actual benefits [23]. Satisfaction, a synonym for attitudes, has a positive effect on users’ intentions of behaviors [24, 25]. The effects of these factors on users’ motivations for various knowledge sharing in OHCs have not been taken into account in previous studies. Diverse knowledge in OHCs is the foundation of health resource integration. It is not yet clear to the effects of these factors on the different types of knowledge sharing in OHCs. Moreover, the effects of costs on the motivations for sharing general and specific knowledge are inconsistent. These benefit-cost factors that influence general and specific knowledge sharing is essential to the sustainability and success of OHCs. Users will actively participate in share knowledge to create value by weighing perceived benefits and costs. Therefore, in order to investigate the effects of perceived benefits and costs on users’ motivations for general and specific knowledge sharing, this study will focus on addressing the following research questions:

RQ1: Which benefit-cost factors might have an impact on users’ motivations for general and specific knowledge sharing in OHCs?

RQ2: How do benefit-cost factors have on users’ motivations for general and specific knowledge sharing in OHCs?

To answer these research questions, based on social exchange theory and previous studies, we propose a research model including intrinsic benefits, extrinsic benefits, cognitive cost, and executional cost. Intrinsic benefits comprise users’ sense of self-worth and satisfaction. Extrinsic benefits comprise social support, online attention, and reputation. We examine the positive effects of perceived benefits on users’ motivations for general and specific knowledge sharing. We further compare the various effects of these factors on users’ motivations for sharing both general and specific knowledge. To examine the model, we conduct an online survey on Chinese health platforms. According to our findings, the motivation for sharing specific knowledge is positively influenced more by satisfaction and social support compared to the motivation for sharing general knowledge. Online attention has a greater effect on the motivation for sharing general knowledge than it does on the motivation for sharing specific knowledge. In addition, cognitive cost negatively affects knowledge sharing motivation, while having different negative effects on general and specific knowledge sharing. Executional cost negatively affects the motivations for sharing both general and specific knowledge in the same way.

This study has several contributions on the motivations for general and specific knowledge sharing. First, this study extends social exchange theory to knowledge sharing in OHCs from a theoretical contribution perspective. The findings strengthen the understanding of perceived benefits and costs on knowledge sharing. In the context of OHCs, the motivation for knowledge sharing are driven by intrinsic and extrinsic benefits, which reflects that social exchange theory is supported. The benefits and costs of knowledge sharing can enhance the application of social exchange theory in the context of OHCs. Second, this study provides important views for the development of OHCs by specifically distinguishing users’ motivations for sharing general and specific knowledge. From a practical contribution perspective, OHCs should focus on users’ perceived benefits and costs to increase their motivations for various types of knowledge sharing. Third, this study adds to the body of literature on the costs for knowledge sharing in OHCs. Users’ motivation to share knowledge can be hampered by costs. There are differences in the negative effects of cognitive costs on users’ motivations to share both general and specific knowledge. This study provides guidance to OHCs managers who should work to reduce the cognitive and executional costs associated with the motivations for general and specific knowledge sharing.

The remainder of this study is arranged as follows. Section 2 introduces knowledge types in OHCs, the motivation for knowledge sharing, and the theoretical background. We discuss the developed hypotheses and detailed methods in Section 3 and Section 4. Section 5 display the findings of the data analyses. Section 6 presents the theoretical and practical implications and limitations. Section 7 summarizes our conclusions.

## 2 Literature review and theoretical background

### 2.1 Knowledge types

Knowledge comprises general and specific knowledge that has been shared in a variety of ways. Contrary to specific knowledge, which is significant throughout the context of communities but peculiar to users in a particular setting, general knowledge is broad, simple to acquire, and typically explicit [26]. General knowledge includes free health articles and specific knowledge includes the number of online consultations for health services [7]. In OHCs, users can submit health consultations and share comments on online health services [27]. To attract online attention from others, users share health articles and register personal information on an online platform. Previous studies have examined the effects of factors on the sharing of both general and specific knowledge [15]. The sustainability of OHCs depend heavily on these benefit-cost variables that affect general and specific knowledge sharing. However, there is a gap in research that specifically examines how the benefits and costs on online health consultations and sharing of comments in OHCs motivate the dissemination and sharing of both general and specific knowledge among users. In this study, general knowledge, such as knowledge on health services, articles and comments on experiences, is a type of public health knowledge. Specific knowledge, such as knowledge on users’ emotion and attitude information, treatments, and medical experiences, is a type of personal health knowledge.

### 2.2 The motivation for knowledge sharing

Knowledge share in OHCs is motivated by a variety of circumstances. Typically, people desire extrinsic and intrinsic benefits for knowledge sharing [10]. The intrinsic and extrinsic benefits for the knowledge sharing in online communities have been explored in the existing studies. For example, Lai and Chen [12] determined that intrinsic motivations significantly affect posters’ knowledge-sharing intentions, while extrinsic motivations significantly affect lurkers’ knowledge-sharing intentions. In the context of OHCs, Zhang et al. [13] identified the effects of intrinsic and extrinsic motivations on knowledge-sharing intentions. Reputation as an external motivation positively affects knowledge sharing [11]. Zhang and Liu [28] integrated social exchange theory and commitment-trust theory to discuss the effects of antecedents on continuous knowledge sharing in OHCs. Empirical research has verified that expected extrinsic benefits have a significant positive effect on knowledge sharing [29]. Intrinsic and extrinsic motivations are key factors influencing knowledge sharing. A sampling of previous studies on knowledge-sharing motivation is presented in Table 1.

**Table 1:** A sampling of previous studies on knowledge sharing motivation.

Number	Context	Independent variables	Dependent variables	Reference
**1**	Electronic networks of practice	Reputation; Enjoy helping, Centrality; Self-rated expertise; Tenure in the field; Commitment; Reciprocity	Knowledge contribution	[11]
**2**	Origanizational climate	Anticipated extrinsic rewards; Anticipated reciprocal relationships; Sense of self-worth; Subjective norm; Organizational climate	Attitude and intention to sharing knowledge (explicit knowledge; implicit knowledge)	[22]
**3**	Electronic knowledge repositories	Knowledge self-efficacy; Enjoyment in helping others; Reciprocity; Organizational reward; Codification effort; Loss of knowledge power	Electronic knowledge repositories usage by knowledge contributions	[10]
**4**	Online feedback system	Enjoyment in helping other consumers; Enjoyment in influencing the company; Economic reward; Cognitive cost; Executional costs	Intention of information contributions	[31]
**5**	Virtual communities	Reputation; Social interaction; Trust; Identification; Reciprocity; Shared language; Altruism	Knowledge sharing behavior (quality; quantity)	[21]
**6**	Online game user communities	Expected relational benefits; Expected intrinsic benefit; Expected extrinsic benefit; Social Tie; Social trust; Shared goals	Attitude and intention toward innovation-conducive knowledge sharing	[32]
**7**	Social Q&A site	Reputation; Social engagement; Enjoyment; Reciprocity; Empathy; Altruism; Personal gain; Self-enjoyment; Self-efficacy	Motivation of health answerers for sharing information	[33]
**8**	Facebook groups	Reputation; Expected relationship; Sense of self-worth; Subjective norm	Attitude toward knowledge sharing, Intention to share knowledge	[34]
**9**	Online communities	Reputation; Reciprocity; Enjoyment in helping others; Knowledge self-efficacy; Offline activities; Enjoyability; Perceived moderator’s enthusiasm	Intention to share knowledge	[12]
**10**	Social commerce sites	Reputation; Enjoyment of helping; Reciprocity; Out-degrees’ Post; In-degrees’ feedback; Customer tenure; Customer expertise	Customer information sharing behavior	[35]
**11**	Online test communities	Self-efficacy; Anticipated extrinsic rewards; Norm of reciprocity; Anticipated reciprocal relationship	Knowledge sharing behavior	[30]
**12**	Online health communities	Reputation; Sense of self-worth; Social support; Face concern; Executional costs; Cognitive costs	Knowledge sharing behavior (general and specific)	[15]
**13**	Online health communities	Reputation; Reciprocity; Knowledge self-efficacy; Altruism; Empathy	Knowledge sharing intention	[13]

However, despite receiving benefits from knowledge sharing on online platforms, users face challenges and incur costs when sharing knowledge, which comprise time, effort, and cognitive aptitude [30]. Few studies on users’ intrinsic and extrinsic motivations for knowledge sharing in OHCs have been found from the benefit-cost perspective. The negative effect of cognitive cost on knowledge sharing has not been verified in previous studies. This study attempts to bridge the gap to explore the effects of various costs on users’ motivations for sharing both general and specific knowledge in OHCs.

### 2.3 Social exchange theory

Social exchange theory [36] explains personal behaviors in the process of social change from the perspective of social psychology, which comprise information technology adoption [37], consumer behaviors [24, 35], and knowledge sharing [10, 29]. The purpose of social exchange theory is to clarify the significance of perceived benefits and costs in the social communications process. The concept of intentions is to maximise benefits while minimising costs, following social exchange theory. Previous studies have identified that the sense of self-worth and satisfaction as intrinsic benefits may positively affect participation in online communities [15, 22, 38, 39]. However, previous studies are not completely understood the different effects of benefits and costs on the motivations for knowledge sharing. Although social exchange theory has been used in some research to examine the direct effects of costs and benefits on knowledge sharing in OHCs, it is unable to explain the different effects of benefit-cost factors on the motivations for general and specific knowledge. Therefore, it is imperative to unlock the crucial factors component that connects the benefits and costs for sharing knowledge. Based on social exchange theory, this study intends to explain the effects of benefit-cost factors on the motivations for general and specific knowledge sharing in OHCs. Online attention, reputation, and social support as extrinsic benefits may positively affect the motivation for knowledge sharing in OHCs. The sense of self-worth and satisfaction as intrinsic benefits in OHCs may have positive effects on the motivations for general and specific knowledge sharing. Costs are generated in the process of knowledge sharing, including two forms of executional and cognitive costs.

## 3 Research model

Online communities have limited utility without extensive knowledge [30]. OHCs are online communities with a focus on health knowledge. According to social exchange theory, people assess benefits and costs during social interactions. The assessment of benefits focuses on perceived intrinsic and extrinsic reward responses, whereas the evaluation of costs focuses on cognitive and executional costs associated with the processing of knowledge sharing. This study distinguishes between the motivations for general and specific knowledge sharing in OHCs. To examine how benefit-cost factors affect users’ motivations for sharing both general and specific knowledge in OHCs, this study proposes a research model with the following components: the sense of self-worth, satisfaction, reputation, social support, and online attention, cognitive and executional cost.

### 3.1 Intrinsic benefits

The sense of self-worth is the extent to which they think their knowledge sharing contributes to online communities. People become confident in their social status and values when they discover the benefits of knowledge sharing [40]. Users with high levels of self-worth are normally more inclined to engage in sharing behaviours than those with low levels of self-worth. Previous studies have identified that knowledge sharing and self-worth are positively correlated [22]. Engaging in knowledge sharing in OHCs can help users receive benefits on the sense of self-worth [15]. Sharing specific knowledge allows users to experience a sense of accomplishment and self-worth in helping others [41]. Users attempt to demonstrate their abilities and gain a sense of self-worth by sharing general knowledge [42]. Users who gain self-worth will contribute information and share knowledge [43]. However, it is not enough to consider the effects of the sense of self-worth on knowledge sharing. Some of the knowledge in OHCs relates to health expertise. We must thoroughly explore how the sense of self-worth affect the motivation for various knowledge sharing. Thus, we put forward the following hypotheses:

***Hypothesis 1a*: *Sense of self-worth positively affects the motivation for general knowledge sharing*.**
***Hypothesis 1b*: *Sense of self-worth positively affects the motivation for specific knowledge sharing*.**

Satisfaction refers to the result of users’ expected rewards compared with the actual benefits of participation behaviors. Previous studies have demonstrated that satisfaction positively affects the intentions to continue using information systems [25]. As an emerging factor in understanding attitudes [23], satisfaction is similar to positive attitudes and promotes online communities to establish long-term relationships among users [24, 38, 39]. Satisfaction, which is an intrinsic motivation, is a predictor of knowledge-sharing motivation [44, 45]. General knowledge, such as online reviews, has an impact on users’ confidence and satisfaction with OHCs [46]. Knowledge on users’ diseases is considered as specific knowledge [15]. Users are willing to exchange specific knowledge with those who share their ailments to discuss treatment experiences [19]. Based on the above studies, the motivation for sharing both general and specific knowledge in OHCs is driven by satisfaction. Thus, we put forward the following hypotheses:

***Hypothesis 2a*: *Satisfaction positively affects the motivation for general knowledge sharing*.**
***Hypothesis 2b*: *Satisfaction positively affects the motivation for specific knowledge sharing*.**

### 3.2 Extrinsic benefits

In an environment where knowledge is plentiful, online attention is a scarce benefit [20]. Due to the perceived benefits, users are inclined to compete for online attention. In this study, online attention is regarded as an extrinsic benefit. Users search for opportunities to express themselves to attract online attention [47]. Previous studies have shown that online knowledge can attract the interest of other users [48]. Online attention affects public information sharing on social media [49]. In the context of online platforms, users compete online attention through frequent knowledge innovation and sharing [50]. Shifting users’ attention to knowledge accuracy can improve the quality of knowledge that users share [51]. General knowledge is accurate information that is publicly accessible and used for health education and recommendations in OHCs [7]. Information on users’ emotional experiences is considered as specific knowledge in OHCs [15]. Users tend to share emotional experiences with others through online platforms, such as sharing memories and telling their own stories [52]. Users who attract online attention may be stimulated to share general and specific knowledge. Thus, we propose the following hypotheses:

***Hypothesis 3a*: *Online attention positively affects the motivation for general knowledge sharing*.**
***Hypothesis 3b*: *Online attention positively affects the motivation for specific knowledge sharing*.**

Reputation is a benefit that extends beyond financial reward and helps users in gaining status and respect in online communities [53–55]. Individuals who believe that sharing knowledge on online platforms enhances their reputations [11]. Individuals establish reputations by sharing their insightful knowledge and consulting experiences [21, 56]. Reputation is an important factor influencing individuals’ intentions and behaviors [57]. Reputation as an extrinsic motivation may have a positive effect on users’ sharing of both general and specific knowledge [15]. Sharing general knowledge positively affects sharing specific knowledge through the mediating role of online reputation [7]. In this study, the same claims about users’ reputation in OHCs are made. Reputation is an extrinsic benefit that may affect users’ motivation for general and specific knowledge sharing. Thus, we put forward the following hypotheses:

***Hypothesis 4a*: *Reputation positively affects the motivation for general knowledge sharing*.**
***Hypothesis 4b*: *Reputation positively affects the motivation for specific knowledge sharing*.**

Social support comprises the support of informational, tangible, emotional, social network, and esteem [58]. Social network impacts users’ health through the pathways of social support [59]. Prayer and spiritual supports are an integral dimension that underlines other social support types in OHCs [60, 61]. Online communities provide safe and relatively anonymous forums for users to communicate on sensitive and potentially stigmatizing topics [34, 62]. Social support is detail on specific health services that users can consult and deal with chronic illnesses [63]. Expressive information in OHCs is actually cathartic and beneficial [64]. Social support facilitates better illness recovery and treatment compliance [65]. Through helpful and encouraging conversation, social support can help people regain emotional equilibrium [66]. In the context of OHCs, users who receive social support feel less isolated and are willing to share knowledge about illnesses and treatment experiences with others. Experiences of users are distinctive and specific knowledge, and social support from specific knowledge boosts users’ confidence and reduces their anxieties and fears [15, 67]. Thus, we put forward the following hypotheses:

***Hypothesis 5a*: *Social support positively affects the motivation for general knowledge sharing*.**
***Hypothesis 5b*: *Social support positively affects the motivation for specific knowledge sharing*.**

### 3.3 Costs

Costs play a significant role in driving knowledge sharing [10, 68]. Social exchange theory states that knowledge will be shared in online communities if the benefits outweigh the costs [22]. In the context of OHCs, costs associated with users sharing knowledge include cognitive and executional costs [15]. Cognitive costs are the emotional expenditure needed to complete something. Before physically reacting to knowledge, people analyse it cognitively [69]. Cognitive costs in existing studies are the consumptions of cognitive resources in the process of stressful events [70]. Sometimes if knowledge presented in OHCs is inaccurate and biased, the knowledge may be stressful for users and lead to worse outcomes [1]. Users must incur cognitive costs in order to judge the objectivity and accuracy of knowledge. Cognitive costs may decrease users’ willingness to share both general and specific knowledge. Executional costs are the time, effort, and resources used to carry out activities. People typically display the inhibition of behaviours when sharing knowledge that requires executional costs [31]. Based on social exchange theory, users should examine the costs and benefits for sharing knowledge before publishing health knowledge in OHCs [71]. Users suffer executional costs while entering and posting both general and specific knowledge [15]. In the context of OHCs, users must put up the time and efforts to share their general and specific knowledge. Thus, we put forward the following hypotheses:

***Hypothesis 6a*: *Cognitive cost negatively affects the motivation for general knowledge sharing*.**
***Hypothesis 6b*: *Cognitive cost negatively affects the motivation for specific knowledge sharing*.**
***Hypothesis 7a*: *Executional cost negatively affects the motivation for general knowledge sharing*.**
***Hypothesis 7b*: *Executional cost negatively affects the motivation for specific knowledge sharing*.**

**Fig 1:**
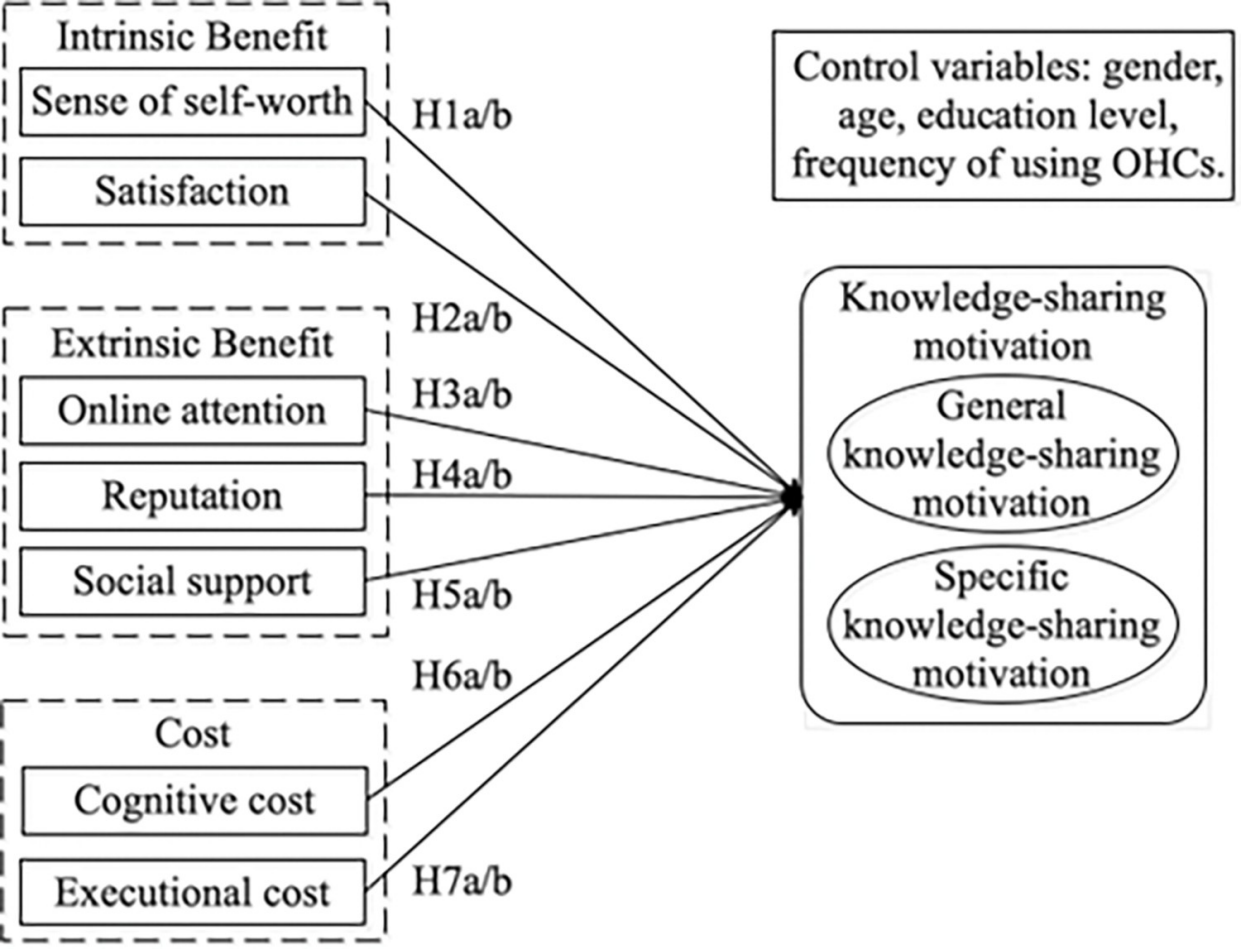
Research model.

Based on the aforementioned hypotheses, our research model is shown in Fig 1.

### 3.4 Control variables

Control variables help improve the internal validity of results. To ensure the reliability and validity of results, researchers can reduce threats to internal validity and interpret results with greater confidence by controlling other potential factors. In this study, users who are physicians and patients asked to share knowledge in OHCs. According to previous studies of controlling individual differences [13, 15, 43], our model comprises four control variables (gender, age, education level, and frequency of OHC usage), which were used to examine users’ characteristics and take the differences among users in OHCs into account.

## 4 Method and measurements

### 4.1 Instrument development

Items in this study were compiled and modified from validated instruments in previous studies to test the proposed hypotheses. A three-item scale was adopted from Shiau et al. [24] to measure satisfaction, and a four-item scale used by Shen et al. [20] was applied to measure online attention. A three-item scale used by Bock et al. [22] was applied to measure the sense of self-worth. We combined the approaches of Yan et al. [15] and Zhang et al. [13] to measure general and specific knowledge-sharing motivations. We used the scale developed by Tong et al. [31] to measure cognitive and executional costs. Social support and reputation were examined according to Yan et al. [15]. All items are listed in S1 File. We used a seven-point Likert scale with anchors of strongly disagree (1) and strongly agree (7).

This study used an expert review and a pilot test to identify ambiguous items. First, 10 experts were invited to discuss the questionnaire and review it. There were five experts in the first group, and they were familiar with empirical study methods. These experts evaluated the context and pointed out unclear items. The second group of experts consisted of five other reviewers with rich experience in conducting treatments in well-known Chinese hospitals. This group evaluated the feasibility of the items. Second, the questionnaire was distributed to 20 respondents from various OHCs. The context and framework of items were suggested by the respondents. Finally, we removed four items that did not represent good construct validity and revised ambiguous items.

### 4.2 Data collection

The formal questionnaire was collected anonymously through a survey website (https://www. wjx.cn/) in China. The website is an online survey platform, providing services on questionnaire design and data collection. We randomly distributed our questionnaires on some online health platforms, such as Sweet Home (bbs.tnbz.com), Good Doctor Online (haodf.com), and DXY (bbs.dxy.cn). Sweet Home is an online community for patients with diabetes to exchange disease knowledge. Through Good Doctor Online, numerous doctors can provide online health services directly to patients. DXY is a digital healthcare platform that connects doctors, patients, hospitals, researchers, and companies. Users share professional knowledge, accumulate comprehensive health information, and acquire high-quality digital health services through DXY. These OHCs are popular health knowledge sharing sites in China.

Individual who had used OHCs could participate in the formal survey. To motivate users’ participations, we promised that each respondent would receive RMB 10 as compensation. The anonymous collection data took place from October to November 2019. All participants provided informed consent before filling in the questionnaire. This study conformed to the principles outlined in the Declaration of Helsinki. Response time for each questionnaire was checked, while questionnaires with completion times significantly lower than the average time were considered as invalid. Finally, a total of 549 questionnaires were received, and 137 invalid questionnaires were discarded. There were 412 valid questionnaires and the recovery rate was 0.75.

Table 2 summarizes the demographic information of the 412 respondents. A total of 56.3% of the respondents were female, and 43.7% were male. Consistent with previous literature, female respondents were slightly more than male respondents. The participants ranged in age from 26 to 40 years and were mostly young. Of the participants sharing knowledge in OHCs, 362 were college graduates. Moreover, 51.7% of the participants indicated that they normally sought health knowledge through OHCs when needed, and only 6.3% of the participants used online platforms nearly daily.

**Table 2:** Descriptive statistics of respondents’ characteristics.

Measure	Item	Frequency	Percentage
Gender	Male	180	43.7%
	Female	232	56.3%
Age	18–25	68	16.5%
	26–40	306	74.3%
	41–50	33	8.1%
	51 or above	5	1.2%
Education level (students and graduates)	High school (or below)	6	1.5%
	College	362	87.9%
	Master (or above)	44	10.7%
Frequency of using OHCs	Almost every day	26	6.3%
	An average of 2–3 times a week	170	41.3%
	When needed	213	51.7%
	Rarely	3	0.7%

## 5 Results

### 5.1 Measurement model analysis

We used AMOS to inspect the construct reliability and convergent and discriminant validity of the measurement model [72]. Table 3 reveals the confirmatory factor analysis results. All constructs indicated good reliability. The constructs’ Cronbach’s α values of all constructs exceeded the standard level of 0.7. The values of composite reliability (CR) of each construct also exceeded 0.7, which indicates good reliability. Factor loadings were over the recommended standard of 0.7 to ensure convergent validity. The average variance extracted (AVE) values of constructs were calculated through the factor loading and the results of all AVE values were over 0.5.

**Table 3:** Confirmatory factor analysis results of the measurement model.

Construct	Item	SE	AVE	CR	Cronbach’s *α*
General knowledge-sharing motivation (GKSM)	GKSM1	.781	.672	.891	.714
	GKSM2	.823			
	GKSM3	.878			
	GKSM4	.794			
Specific knowledge-sharing motivation (SKSM)	SKSM1	.707	.685	.866	.762
	SKSM2	.884			
	SKSM3	.879			
Sense of self-worth (SSW)	SSW1	.767	.635	.770	.757
	SSW2	.815			
	SSW3	.807			
Online attention (OA)	OA1	.884	.778	.792	.773
	OA2	.848			
	OA3	.913			
Social support (SS)	SS1	.948	.743	.806	.870
	SS2	.881			
	SS3	.744			
Reputation (REP)	REP1	.893	.773	.809	.773
	REP2	.914			
	REP3	.890			
	REP4	.816			
Cognitive cost (CC)	CC1	.974	.779	.941	.767
	CC2	.857			
	CC3	.808			
Executional cost (EC)	EC1	.805	.654	.836	.833
	EC2	.791			
	EC3	.807			
	EC4	.831			
Satisfaction (SAT)	SAT1	.881	.941	.842	.741
	SAT2	.928			
	SAT3	.942			

Table 4 reveals the correlations between the constructs. To verify the discriminant validity, we determined that the correlations between constructs were less than the square root of the AVE value. The main diagonal values were over 0.7 and exceeded the correlations between any pair of constructs. All values indicate that our research model has sufficient discriminant validity.

**Table 4:** Correlations between constructs.

Constructs	Mean	SD^a^	GKSM	SKSM	SSW	OA	SS	REP	CC	EC	SAT
GKSM	5.03	1.13	**.821** ^b^								
SKSM	4.32	1.18	.622^c^	**.827**							
SSW	5.61	.878	.206	.233	**.797**						
OA	4.96	.973	.313	.464	.326	**.882**					
SS	5.64	.622	.219	.365	.204	.264	**.862**				
REP	4.94	1.22	.314	.491	.265	.389	.344	**.879**			
CC	2.46	.808	-.130	-.246	-.120	-.115	-.154	-.109	**.882**		
EC	3.49	.687	.324	.512	.178	.228	.245	.251	-.300	**.809**	
SAT	5.77	.640	.268	.418	.272	.239	.298	.273	-.201	.398	**.917**

^a^standard deviation

^b^the diagonal numbers are the square root of AVE

^c^off-diagonal elements are the correlations among constructs.

Table 5 reveals that the model fit index was acceptable. The confirmatory factor analysis indicated that our research model fits the collected data well (*χ*^2^ = 714). The ratio between *χ*^2^ and the degree of freedom (*χ*^2^/df) was 1.493. The goodness-of-fit index (GFI) was 0.929, the Tucker-Lewis index (TLI) was 0.942, and the comparative fit index (CFI) was 0.955. The incremental fit index (IFI) was 0.956, and the adjusted GFI (AGFI) was 0.901. The root mean square error of approximation (RMSEA) was 0.035. The model fitting indexes mostly overstepped the recommended levels, which illustrates that the measurement model had a good fit with the collected data.

**Table 5:** Goodness-of-fit assessments.

Goodness-of-fit measures	*χ*^2^/df	GFI	AGFI	TLI	CFI	IFI	RMSEA
Goodness-of-fit ranges	1~3	> .900	> .900	> .900	> .900	> .900	< .050
SEM model	1.493	.929	.901	.942	.955	.956	.035

### 5.2 Structural model

**Fig 2:**
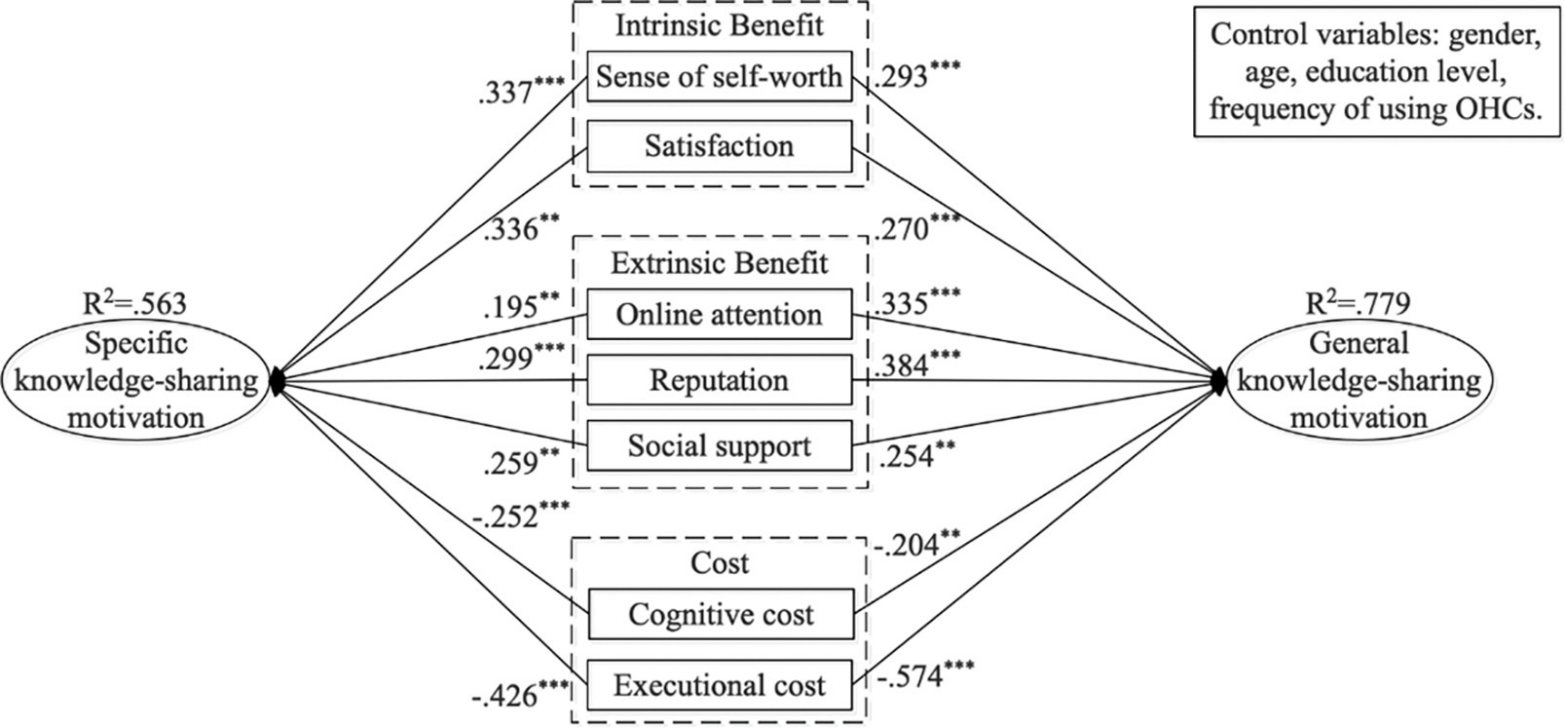
Path coefficients and significance levels.

We used the structural equation model (SEM) technique to analyze the effects of benefit-cost factors on the motivations for knowledge sharing. To test the structural model, we used AMOS to analyze our proposed hypothesis. As shown in Table 6 and Fig 2, the sense of self-worth (β = 0.293, p < 0.001) and satisfaction (β = 0.270, p < 0.001) are determinants of the motivation for general knowledge sharing. Intrinsic benefits positively affect the motivation for general knowledge sharing. Similarly, sense of self-worth (β = 0.337, p < 0.001) and satisfaction (β = 0.366, p < 0.01) positively influence the motivation for specific knowledge sharing. In the context of extrinsic benefits, online attention (β = 0.335, p < 0.001) and reputation (β = 0.384, p < 0.001) have significant effects on the motivation for general knowledge sharing. Moreover, online attention (β = 0.195, p < 0.01) and reputation (β = 0.299, p < 0.001) are related to the motivation for specific knowledge sharing. Social support positively affects the motivation for general and specific knowledge sharing. H5a (β = 0.254, p < 0.01) and H5b (β = 0.259, p < 0.01) were supported. Cognitive and executional costs negatively affect the motivations for two types of knowledge sharing. Thus, H6a (β = -0.204, p < 0.01), H6b (β = -0.252, p < 0.001), H7a (β = -0.574, p < 0.001), and H7b (β = -0.426, p < 0.001) were supported.

**Table 6:** Results of the hypothesis testing.

Hypothesis	Std. Beta	Std. Error	*t*-value	*p*-value
*H1a*: *Sense of self-worth positively affects the motivation for general knowledge sharing*.	.293	.042	6.976	*** ^a^
*H1b*: *Sense of self-worth positively affects the motivation for specific knowledge sharing*.	.337	.098	3.439	***
*H2a*: *Satisfaction positively affects the motivation for general knowledge sharing*.	.270	.058	4.655	***
*H2b*: *Satisfaction positively affects the motivation for specific knowledge sharing*.	.366	.068	5.382	** ^b^
*H3a*: *Online attention positively affects the motivation for general knowledge sharing*.	.335	.070	4.786	***
*H3b*: *Online attention positively affects the motivation for specific knowledge sharing*.	.195	.065	2.951	**
*H4a*: *Reputation positively affects the motivation for general knowledge sharing*.	.384	.055	4.329	***
*H4b*: *Reputation positively affects the motivation for specific knowledge sharing*.	.299	.062	4.823	***
*H5a*: *Social support positively affects the motivation for general knowledge sharing*.	.254	.079	3.342	**
*H5b*: *Social support positively affects the motivation for specific knowledge sharing*.	.259	.095	2.726	**
*H6a*: *Cognitive cost negatively affects the motivation for general knowledge sharing*.	-.204	.038	5.368	**
*H6b*: *Cognitive cost negatively affects the motivation for specific knowledge sharing*	-.252	.047	5.248	***
*H7a*: *Executional cost negatively affects the motivation for general knowledge sharing*.	-.574	.159	3.618	***
*H7b*: *Executional cost negatively affects the motivation for specific knowledge sharing*.	-.426	.065	6.552	***

^a^ *** p<0.001

^b^ ** p <0.01

We separately performed regression on the various effects of factors on two motivations for knowledge sharing. We used a t-test to assess the difference among the effects of antecedent variables on the motivation for two types of knowledge sharing [73]. We used bootstrapping to assess the significance of each path through t-tests [74]. The t-values for the contributions of sense of self-worth and reputation to the motivations for general and specific knowledge sharing were significant. The t-values for the contribution of satisfaction, online attention, and social support to the motivations for general and specific knowledge sharing were different in significance. the t-values for the contribution of cognitive costs to the motivations for general and specific knowledge sharing were different in significance, which differs from previous studies [15]. However, the t-values for the contribution of executional costs to the motivations for general and specific knowledge sharing were significant, respectively.

Furthermore, the standardized root means square residual (SRMR) was measured to assess the fit between the empirical and theoretical results. The value of SRMR in this study was 0.048, which is within the recommended range. Thus, the structural model has a good fit. Fig 2 shows that all antecedents that directly affected the motivation for general knowledge sharing accounted for 77.9% of its variance, and all antecedent variables that directly affected the motivation for specific knowledge sharing accounted for 56.3% of its variance. To examine the difference in general and specific knowledge-sharing motivation, this study used an alternative model in which the path coefficients of the general and specific knowledge-sharing motivation are similar.

### 5.3 Common method bias

The collected data in this study are self-reported data from all respondents and may have common method biases [75–77]. First, we conducted a Harmon one-factor test to evaluate common method bias. All constructs accounted for 72.8% of the total variance, with the first factor explaining the highest variance, 29.4%. Second, we added a common method in the research model, and the items comprised all the principal constructs’ items and calculated each of the item’s variances that were substantively explained by the constructs in our research model. Table 7 shows that none of the method factor loadings were significant and that the average substantively explained variance of the items was 0.668, while the average method-based variance was 0.008. Due to the small and insignificant method-based variance, common method biases were not serious problems for the collected data [75, 78].

**Table 7:** Common method bias analysis.

Construct	Indicator	Substantive factor loading (R1)	R1^2^	Method factor loading (R2)	R2^2^
General knowledge-sharing motivation (GKSM)	GKSM1	.769***^a^	.591	.052	.003
	GKSM2	.835***	.697	.038	.001
	GKSM3	.866***	.750	.027	.001
	GKSM4	.802***	.643	.182	.033
Specific knowledge-sharing motivation (SKSM)	SKSM1	.755***	.570	.088	.008
	SKSM2	.871***	.759	.094	.009
	SKSM3	.796***	.634	.059	.003
Sense of self-worth (SSW)	SSW1	.733***	.537	.048	.002
	SSW2	.869***	.755	.038	.001
	SSW3	.873***	.762	.025	.001
Online attention (OA)	OA1	.751***	.564	.027	.001
	OA2	.735***	.540	.171	.029
	OA3	.887***	.787	.032	.001
Social support (SS)	SS1	.856***	.733	.085	.007
	SS2	.843***	.711	.042	.002
	SS3	.739***	.541	.096	.009
Reputation (REP)	REP1	.815***	.664	.069	.005
	REP2	.829***	.687	.086	.007
	REP3	.751***	.564	.133	.018
	REP4	.736***	.542	.063	.004
Cognitive cost (CC)	CC1	.877***	.769	.087	.008
	CC2	.763***	.582	-.019	.001
	CC3	.728***	.530	-.023	.001
Executional cost (EC)	EC1	.859***	.738	.033	.001
	EC2	.788***	.621	-.138	.019
	EC3	.813***	.661	-.043	.002
	EC4	.849***	.721	.014	.001
Satisfaction (SAT)	SAT1	.849***	.738	.064	.004
	SAT2	.906***	.840	.089	.008
	SAT3	.928***	.880	.248	.062
Average		.815	.668	.072	.008

^a^ *** p <0.001

### 5.4 Robustness check

We conducted a robustness check to test the validity of our results. Another tool, SmartPLS, was used to test our research model and examine the robustness of our previous results [73]. Table 8 shows that the hypothesis testing results using SmartPLS were consistent with the previous results using AMOS.

**Table 8:** Results of hypothesis testing using SmartPLS.

Hypothesis	Std. Beta	Std. Error	*t*-value	*p*-value	Outcomes
H1a	.230	.093	8.214	*** ^a^	Supported
H1b	.148	.088	4.625	***	Supported
H2a	.595	.044	6.230	***	Supported
H2b	.380	.061	13.523	***	Supported
H3a	.440	.067	11.892	***	Supported
H3b	.237	.031	7.645	***	Supported
H4a	.479	.115	4.165	***	Supported
H4b	.335	.178	4.295	***	Supported
H5a	.368	.106	6.033	***	Supported
H5b	.260	.054	4.815	***	Supported
H6a	-.132	.134	3.882	***	Supported
H6b	-.231	.178	2.817	** ^b^	Supported
H7a	-.259	.077	3.364	***	Supported
H7b	-.316	.028	5.097	***	Supported

^a^ *** p<0.001

^b^ ** p <0.01

## 6 Discussion

### 6.1 Theoretical implications

This study contains three theoretical implications regarding users’ motivations for both general and specific knowledge sharing in OHCs. First, by explaining the positive effects of perceived benefits, such as satisfaction and online attention, this study enriched the literature on the benefits of knowledge in OHCs. To learn how sharing general and specific knowledge can be motivated by benefits, we expanded on satisfaction and online attention as perceived benefits in the research model. Results enhance the interpretation of benefits that promote knowledge sharing in OHCs.

Second, this study enhances comprehension of the literature on online health knowledge sharing. We provide a novel view of general and specific knowledge in the context of OHCs. Our results contribute to previous studies on the motivations for general and specific knowledge sharing [7, 15]. Satisfaction and social support have more positive effects on the motivation for specific knowledge sharing than on the motivation for general knowledge sharing. However, online attention has a more positive effect on the motivation for general knowledge sharing than on the motivation for specific knowledge sharing.

Third, this study contributes to the existing research on social exchange theory in OHCs. Knowledge sharing motivations are affected by benefits and costs rather than just the benefits. By examining the negative effect of costs on the motivation for knowledge sharing, our results show a clear path for OHCs to inhibit the motivations for sharing both general and specific knowledge. Users’ motivations for general and specific knowledge sharing are negatively related to cognitive and executional costs. In addition, the negative effect of cognitive cost on users’ motivation for sharing specific knowledge is substantially greater than its negative effect on the motivation for sharing general knowledge.

### 6.2 Practical implications

This study contains some practical implications for the development of OHCs. First, our results indicate that users’ satisfaction with OHCs motivates the sharing of specific knowledge. Users are willing to share specific knowledge after receiving social support in OHCs. Satisfaction and social support can significantly contribute to users’ contributions to OHCs, such as sharing treatment experiences, comments for health knowledge, and health status after adopting suggestions. OHCs provide a wealth of specific knowledge for users to communicate and attract users for promoting online health platforms’ continuous growth.

Second, OHCs managers need to make a distinction between cognitive cost and executional cost. Simple interface and content input options can reduce the users’ time consumed in the process of knowledge sharing. The user-friendly interface is essential for increasing motivation to share knowledge. Designers of OHCs can diversify the forms of knowledge sharing, such as voice, video, and graphics. Through the function of content visualization to reduce users’ cognitive costs, specific knowledge can be represented as long diagrams and word clouds.

Third, our results indicate that intrinsic and extrinsic benefits positively affect the motivation for two types of knowledge sharing. Knowledge of treatments and recovery experiences can allow users to gain social support and online attention. We provide design implications for OHCs to facilitate social support better by facilitating supportive communication, supporting network visualization, and mobilization [61]. Designers of OHCs can encourage users to share specific knowledge and set rewards for users, such as gifts and emotional rewards. Moreover, users’ satisfaction with OHCs can be increased by improving the responsiveness of knowledge sharing.

### 6.3 Limitations and future research directions

This study has several limitations that can provide directions for future research. First, we measured constructs from self-reported data and used a cross-sectional survey to collect data. Though the robustness of results was ensured by checking the reliability and validity, future research can measure the effects of the benefit-cost factors on the motivations for general and specific knowledge sharing over time. Second, not every OHC user has been investigated. This study has collected data through an online survey of users from Chinese OHCs. Respondents may have been biased toward individuals who were familiar with OHCs and were willing to understand our online survey. Future studies should consider objective data collection methods and verify whether our research model remains supported. Third, although the data have supported our research model, other significant variables, including trust, reciprocity, and peer recognition, have been examined in other studies to predict knowledge sharing [79, 80]. Future studies can discuss the effects of additional factors on knowledge sharing in OHCs. Various types of OHCs may have various effects on the motivations for sharing both general and specific knowledge. In the future, we will take into account the various effects of benefit-cost aspects on users’ knowledge sharing in different types of OHCs.

## 7 Conclusions

We investigate users’ motivations for general and specific knowledge sharing in OHCs. Based on social exchange theory, antecedents include intrinsic benefits (sense of self-worth, satisfaction), extrinsic benefits (social support, reputation, and online attention), cognitive cost, and executional cost. Our results discover the significant effects of these factors on the motivations for general and specific knowledge sharing. We compared the various effects of antecedents on the motivations for general and specific knowledge sharing in OHCs. In comparison to the motivation for general knowledge sharing, the effects of satisfaction and social support on the motivation for specific knowledge sharing are stronger. online attention has a greater impact on the motivation for general knowledge sharing than it does on the motivation for specific knowledge sharing. The detrimental consequences of cognitive costs on the motivation of two different types of knowledge sharing vary. With regard to various forms of knowledge sharing in OHCs, this study provides theoretical and practical implications. This study contributes to the understanding of the survival and value realization of OHCs by investigating users’ motivations for sharing both general and specific knowledge from a benefit-cost perspective.

## Supporting information

S1 File Appendix of measurement instrument. It shows all items of constructs.
(DOCX)

S2 File The minimal anonymized data used in the calculations of all tables and figures. (CSV)
