# Supplementary material for: The effects of factors on the motivations for knowledge sharing in online health communities: A benefit-cost perspective
Source: PLoS One. 2023 Jun 12;18(6):e0286675. doi: 10.1371/journal.pone.0286675 (PMC10259799; doi:10.1371/journal.pone.0286675)
Supplement: S1 File — It shows all items of constructs. (DOCX) [file pone.0286675.s001.docx]

**Appendix. Measurement instrument.**

| **Construct** | **Items** | **Source** |
| --- | --- | --- |
| General knowledge-sharing motivation (GKSM) | 1. When participating in an OHC, I intend actively to share public information I know. | [13, 15] |
|  | 2. When discussing problems related to the hospital, medicine, and other public issues, I intend to involve in the subsequent interactions. |  |
|  | 3. I intend to spend a lot of time conducting general knowledge sharing activities in OHCs. |  |
|  | 4. I intend to frequently participate in general knowledge sharing activities in OHCs. |  |
| Specific knowledge-sharing motivation (SKSM) | 1. When discussing problems related to treatment experience and other private issues, I intend to involve in the subsequent interactions. | [13, 15] |
|  | 2. I intend to spend a lot of time conducting specific knowledge sharing activities in OHCs. |  |
|  | 3. I intend to frequently participate in specific knowledge sharing activities in OHCs. |  |
| Satisfaction (SAT) | 1. I feel very satisfied with the health knowledge in online health communities. | [24] |
|  | 2. I feel very pleased with OHCs to provide health information I need. |  |
|  | 3. I feel very contented with my treatment experiences provided by OHCs. |  |
| Sense of self-worth (SSW) | 1. My knowledge sharing would help other members in OHCs solve problems. | [22] |
|  | 2. My knowledge sharing would bring positive influence on other members in OHCs. |  |
|  | 3. My knowledge sharing would bring all my facilities into full play and make me more confident. |  |
| Online Attention (OA) | 1. I care about others’ online attitudes toward me. | [20] |
|  | 2. Sharing knowledge with other members in OHCs will make me gain online attention. |  |
|  | 3. I will gain online attention if I have latest health knowledge. |  |
| Reputation (REP) | 1. Sharing knowledge can enhance my reputation in OHCs.  2. I get praise from others by sharing knowledge in OHCs. | [15] |
|  | 3. I feel that knowledge sharing improves my status in OHCs. |  |
|  | 4. I can earn some feedback or rewards through knowledge sharing that represent my reputation and status in OHCs. |  |
| Social support (SS) | 1. Through knowledge sharing in OHCs, I pour out may troubles and feel relaxed. | [15] |
|  | 2. Through knowledge sharing in OHCs, I get comfort and care from other members in the communities. |  |
|  | 3. Through knowledge sharing in OHCs, I get some understanding, help or supports from other members in the communities. |  |
| Cognitive costs (CC) | 1. It is annoying to recall every detailed aspect of my or others’ medical experience in order to share knowledge in OHCs. | [31] |
|  | 2. It is not enjoyable to recall my or others’ medical treatment procedure in order to share knowledge in OHCs. |  |
|  | 3. It is difficult for me to recollect treatment experience and treatment solution. |  |
| Executional costs (EC) | 1. I can’t seem to find the time to share knowledge in OHCs. | [31] |
|  | 2. It is laborious to share knowledge in OHCs. |  |
|  | 3. It takes me too much time to share knowledge in OHCs. |  |
|  | 4. The effort is high for me to share knowledge in OHCs. |  |
